# Genu Recurvatum After Prolonged Bracing for Drop-Foot in a Patient With History of Guillain-Barre Syndrome

**DOI:** 10.7759/cureus.10587

**Published:** 2020-09-22

**Authors:** Panagiotis V Samelis, Panagiotis Kolovos, Flourentzos Georgiou, Christos Loukas, Cathrin Catsouli

**Affiliations:** 1 Orthopaedics, Orthopaedic Research and Education Center, Attikon University Hospital, Athens, GRC; 2 1st Orthopaedic Department, Children’s General Hospital Panagiotis & Aglaia Kyriakou, Athens, GRC

**Keywords:** physis, arrest, guillain-barre, genu, recurvatum, orthosis, drop-foot, osteotomy, cora, brace

## Abstract

A case of unilateral genu recurvatum (GR) in a 15-year-old boy with a history of Guillain-Barre syndrome (GBS) and subsequent bilateral drop-foot is presented. Muscle imbalance of the lower limb and repetitive pressure from prolonged usage of an orthosis to deal with drop-foot may be the causative factors for early partial physeal arrest of his right proximal tibia. The result was a right GR and a shorter right lower limb. A below the tibial tuberosity anterior opening-wedge oblique proximal tibial osteotomy was performed. The deformity was gradually corrected using an Ilizarof circular frame. The center of rotation and angulation of the procedure was placed at the posterior tibial cortex. The procedure was completed uneventfully within four months. Excellent clinical and radiological improvement of the deformity was obtained.

## Introduction

The term genu recurvatum (GR), or back-knee, describes an angular deformity of the knee on the sagittal plane. The affected lower limb presents a hyperextended knee and is shorter than the contralateral. As a result, the posture and the gait of the individual is greatly affected and disabled [1,2].

Various factors may lead to GR [1]. Direct trauma with or without fracture of the proximal tibia, surgical intervention on the proximal tibia, prolonged skeletal tibial traction, osteomyelitis, irradiation, Osgood-Schlatter disease, prolonged immobilization, direct plaster cast or brace pressure on the proximal tibial physis, ligamentous laxity, posterolateral corner insufficiency, gastrocnemius weakness, tumors, cerebrovascular accident and poliomyelitis correlate with the deformity [1-6]. Congenital, hereditary and idiopathic cases have also been reported [1, 7-9]. GR may be osseous, ligamentous or mixed [4,7]. Osseous GR is the result of premature closure of the anterior part of the proximal tibial growth plate, while the posterior part of the physis continues to grow [2,4,7].

GR will pose significant compressive load on the medial tibiofemoral joint and tensile load on the posterolateral corner and the anterior cruciate ligament (ACL), leading to a painful joint [5,10]. GR may predispose to ACL injury in female athletes [5]. It has been reported, that knee hyperextension is an independent predictor of knee stability and function and predisposes to ACL graft impingement and failure of ACL reconstruction [10]. Whatever the cause, GR may lead to symptoms, such as pain or instability of the affected knee, patellofemoral disorders, leg length discrepancy and gait disturbance [1,7].

There is no consensus on the amount of knee hyperextension that should be deemed a pathologic GR [10]. Recurvatum of the knee may be the rule and not the exception, with a mean of +3.1⁰ for men and +5.7⁰ for women [11]. A knee recurvatum up to 15⁰ has been demonstrated to be a normal finding in 40% of healthy controls, is usually symmetric and bilateral (constitutional recurvatum) [7,12]. Dejour supports, that a pathologic GR is always acquired and unilateral [7]. It has been suggested, that a symptomatic GR greater than 5⁰ should be evaluated and treated [1,5]. However, a recurvatum greater than 15⁰ should always be considered as pathologic and should be treated to restore normal mechanical conditions of the knee and to improve the quality of life of the patient [2,12]. An oblique anterior opening-wedge proximal tibial osteotomy is the widely accepted treatment for osseous GR. It can be accomplished either as one-stage procedure, or gradually, using the distraction osteogenesis technique [1,2,12,13].

## Case presentation

A 15-year-old boy presented to the orthopedic outpatient clinic with right GR. He had a history of Guillian-Barre syndrome (GBS) at the age of 10 years, for which he was treated in the Intensive Care Unit (ICU). He stayed in the ICU for two months, most of it, intubated. GBS resolved gradually after several regimens of immunoglobulin and plasmaferesis. On discharge, torso and limb muscle strength had restored to grade M4 (muscle resistance below normal) and M5 (normal resistance), except for the muscles of the anterior compartment of the tibia (M0 to M1, no muscle movement or muscle movement without joint motion respectively). This deficit was bilateral and the patient used ankle-foot orthosis for several months to compensate drop-foot. Some years later, during adolescence, the patient gradually developed a recurvatum deformity of his right knee. He did not recall any serious traumatic event of the right tibia or knee in recent years.

**Figure 1:**
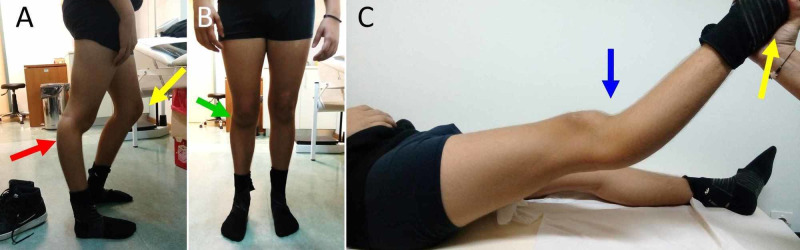
A 15-year-old boy with right knee recurvatum A. The typical posture of the patient with unilateral acquired genu recurvatum: hyperextension of the affected right knee (red arrow) with flexion of the healthy contralateral knee (yellow arrow) due to a shorter right lower limb. B. Slight valgus of the affected right knee (green arrow). C. With the patient supine, lifting the affected lower limb from the heel (yellow arrow) reveals the recurvatum deformity of the right knee (blue arrow).

The patient was walking without support, however, his gait was markedly affected due to bilateral drop-foot and unilateral right GR. In the upright position the patient presented the typical GR-posture. This posture consists of hyperextension of the affected right knee and flexion of the healthy contralateral knee, in an attempt of the patient to compensate limb length discrepancy due to a shorter right lower limb. Slight valgus of the right knee when standing, corrected significantly on passive hyperextension of the knee with the patient supine and was rather a component of the GR-posture and not a major part of the GR osseous deformity. On clinical examination, some medial laxity of the right knee was manifested, compared to the contralateral, however, the tibiofemoral and the patellofemoral joint were more or less stable and asymptomatic, without evidence of major ligamentous deficit (Figure 1).

**Figure 2:**
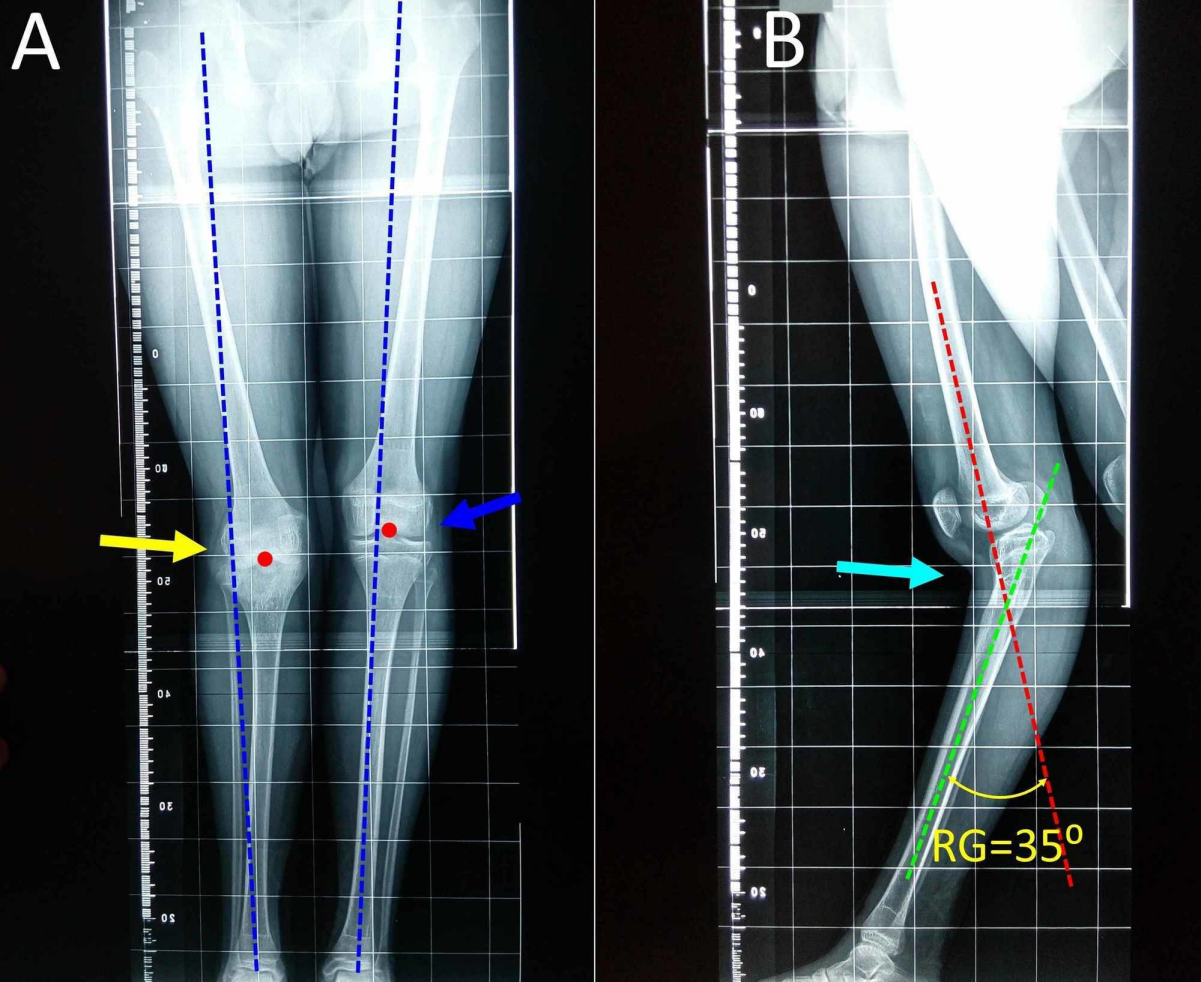
Standing long-limb radiograph of the 15-year-old boy with genu recurvatum A. Anteroposterior view: the mechanical axis (blue line) of the right lower limb (arrow) deviates laterally from knee center (red spot), compared with the contralateral knee (blue arrow), indicating a valgus deformity of the right knee. B. Lateral long-limb weight-bearing view of the right lower limb (arrow): the recurvatum angle (RG) is the angle formed by the intersection of the distal lateral axis of the femur (red line) and the axis of the shaft of the tibia (green line). RG: recurvatum angle

The deformity was further studied using a long-limb standing (weight-bearing) x-ray projection of both limbs (Figure 2). On the anteroposterior view, lateral deviation of the mechanical axis of the right lower limb away from the center of the knee is evident, mainly due to postural and not osseous deformity of the lower limb. On the lateral view of the right lower limb the recurvatum angle (RG, the angle formed between the distal lateral anatomical axis of the right femur and the lateral anatomical axis of the right tibia) was 35⁰ [2,12-15]. A limb length discrepancy due to a 2 cm shorter right tibia was attributed mainly to a shorter anterior tibial cortex compared to the normal posterior cortex and secondarily to early arrest of the proximal tibial growth plate.

Preoperative planning

**Figure 3:**
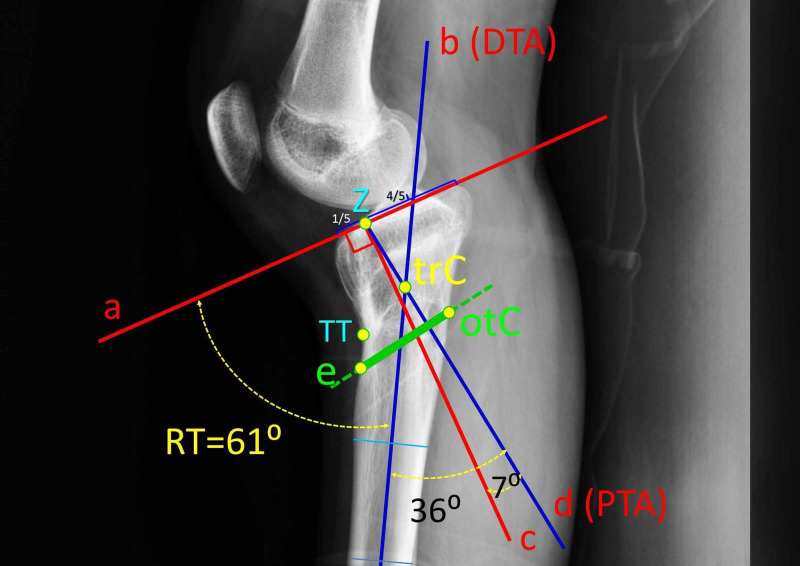
Preoperative planning of the osteotomy Lines a, b, c, d and e are drawn. Line a is the plane of the tibial plateau. Line b is the distal lateral axis of the tibial shaft (DTA). Line c is vertical to line a. It starts at point Z, that separates the anterior 1/5th and the posterior 4/5ths of the tibial plateau. Line d (the proximal tibial axis, PTA) starts from point Z with a posterior inclination of 7⁰ relative to line c. The tilt angle of the right tibial plateau (RT-angle) is formed by the intersection between the line a and line b (DTA). The true center of rotation and angulation (true-CORA, point trC) of the deformity is the intersection between line b (DTA) and line d (PTA). The CORA of the osteotomy (osteotomy-CORA, point otC) is placed on the dorsal tibial cortex, about 1 cm distal to the true-CORA. Line e is the plane of the osteotomy. The osteotomy starts 1 cm below the tibial tubercle (TT) and exits at the osteotomy-CORA (otC).

The first step is to define whether the deformity is osseous, ligamentous or mixed. The tilt angle of the right tibial plateau is measured (RT-angle). This angle represents the osseous deformity of GR (Figure 3). The RT-angle is formed by the intersection between the plane of the tibial plateau (line a) and the distal lateral axis of the right tibia (DTA, line b). The RT-angle is normally 97⁰ (the normal posterior tibial slope is considered 7⁰) [2,12-15]. In the presented case, the RT-angle is 61⁰, a difference from normal of 36⁰ (97⁰-61⁰). This difference is practically equal to the recurvatum angle (RG=35⁰). This means, that the deformity of the patient is pure osseous, without a capsuloligamentous component [2,7]. An anterior opening-wedge oblique proximal tibial osteotomy was decided.

The next step is to determine the center of rotation and angulation (CORA) and the plane of the osteotomy (Figure 3). On the lateral x-ray projection of the tibia, a line (line c) vertical to the line of the tibial plateau (line a) is drawn. Line c starts from the point, that separates the anterior 1/5th and the posterior 4/5ths of the tibial plateau (point Z). This is the point, where the lateral axis of the tibial shaft normally intersects with the tibial plateau [13,15]. Starting from point Z, a line (line d) is drawn, with a posterior inclination of 7⁰ (normal posterior tibial slope) relative to line c. Line d represents the proximal tibial axis (PTA). The intersection between DTA (line b) and PTA (line d) defines the CORA of the deformity (true-CORA, point trC) [13,15].

In this patient, a CORA different from the true-CORA was chosen for the osteotomy (osteotomy-CORA, point otC). The osteotomy-CORA was placed dorsally and distally compared with the true-CORA. Placing the osteotomy-CORA at the posterior tibial cortex aims at correction of the angulation with simultaneous tibial lengthening. Furthermore, the osteotomy-CORA allowed a plane of the osteotomy (line e), which started 1 cm below the tibial tubercle (point TT), and exited at the dorsal tibial cortex. Thus, the osteotomy is far enough from the knee joint, in order to spare an asymptomatic patellofemoral joint from the procedure and to avoid intraarticular passage of the tension wires of the Ilizarof frame (Figure 3) [16].

The procedure

**Figure 4:**
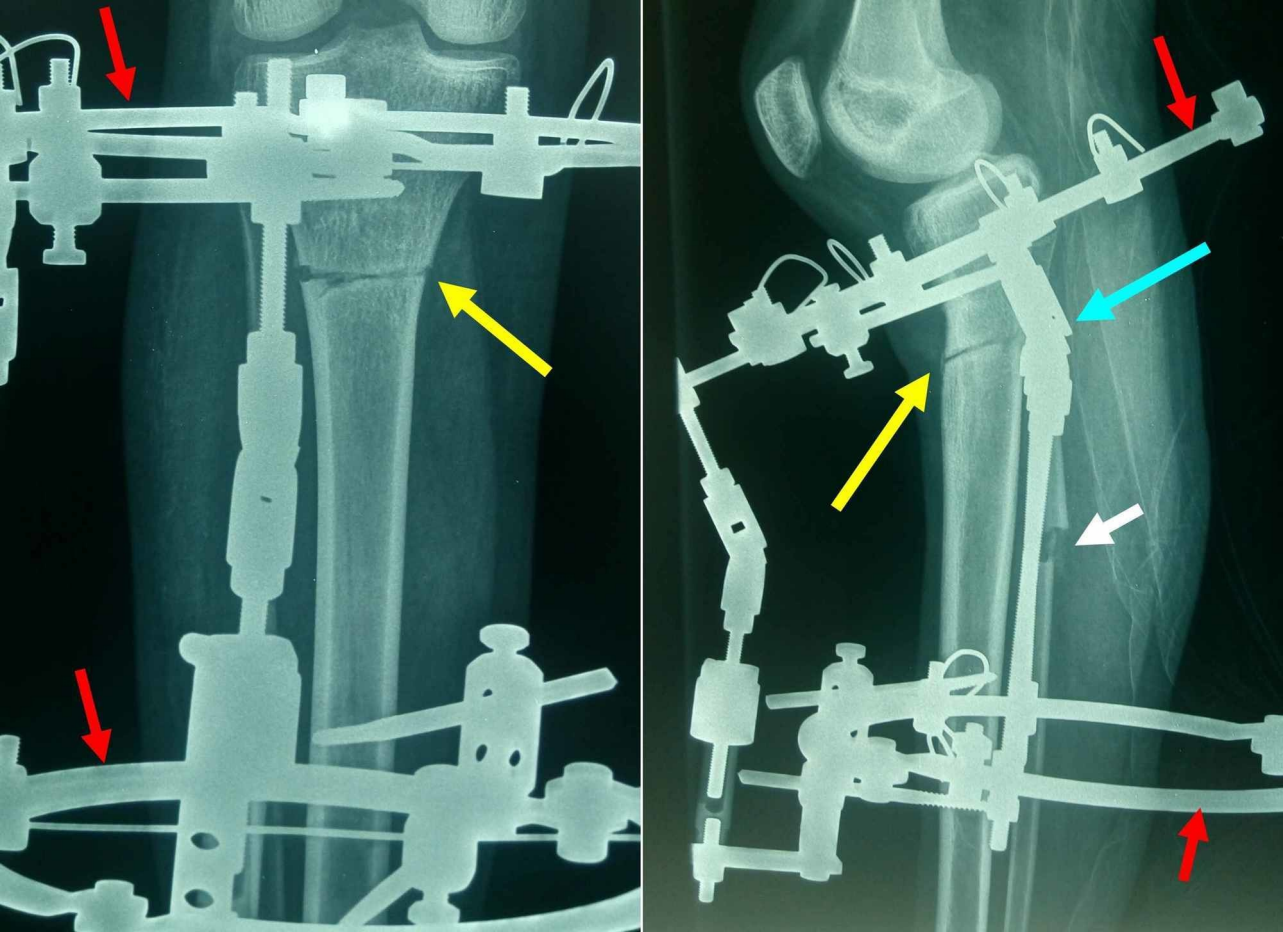
Ilizarof frame and hinge placement relative to the osteotomy level A. Anteroposterior view: the Ilizarof frame consists of two rings (red arrows), placed on either side of the osteotomy (yellow arrow) using tension wires and half-pins. B. Lateral view: the Ilizarof rings (red arrows), the osteotomy (yellow arrow), the fibular osteotomy (white arrow) and hinge placement (blue arrow). Exact placement of the hinges of the fixator according to the osteotomy-CORA is mandatory to achieve simultaneous correction of angulation and limb length deficit.

Aim of the procedure is to correct the osseous GR by restoring the RT-angle. Gradual correction using the Ilizarof method was chosen. An Ilizarof circular frame was applied. One proximal and one distal ring were attached on either side of the planned osteotomy using 2,5 mm tension wires and 6 mm half pins. The plane of the osteotomy was parallel to the tibial plateau and was accomplished by multiple drilling of the tibia through a small (about 1 cm) anterior skin incision below the tibial tuberosity. The hinges of the Ilizarof frame corresponded to the osteotomy-CORA. Finally, fibular shaft osteotomy through a separate incision with resection of a small (0,5-1cm) fragment of the fibula was performed to allow distraction of the construct. All stages of the procedure were monitored by image intensification (Figure 4).

Follow up

**Figure 5:**
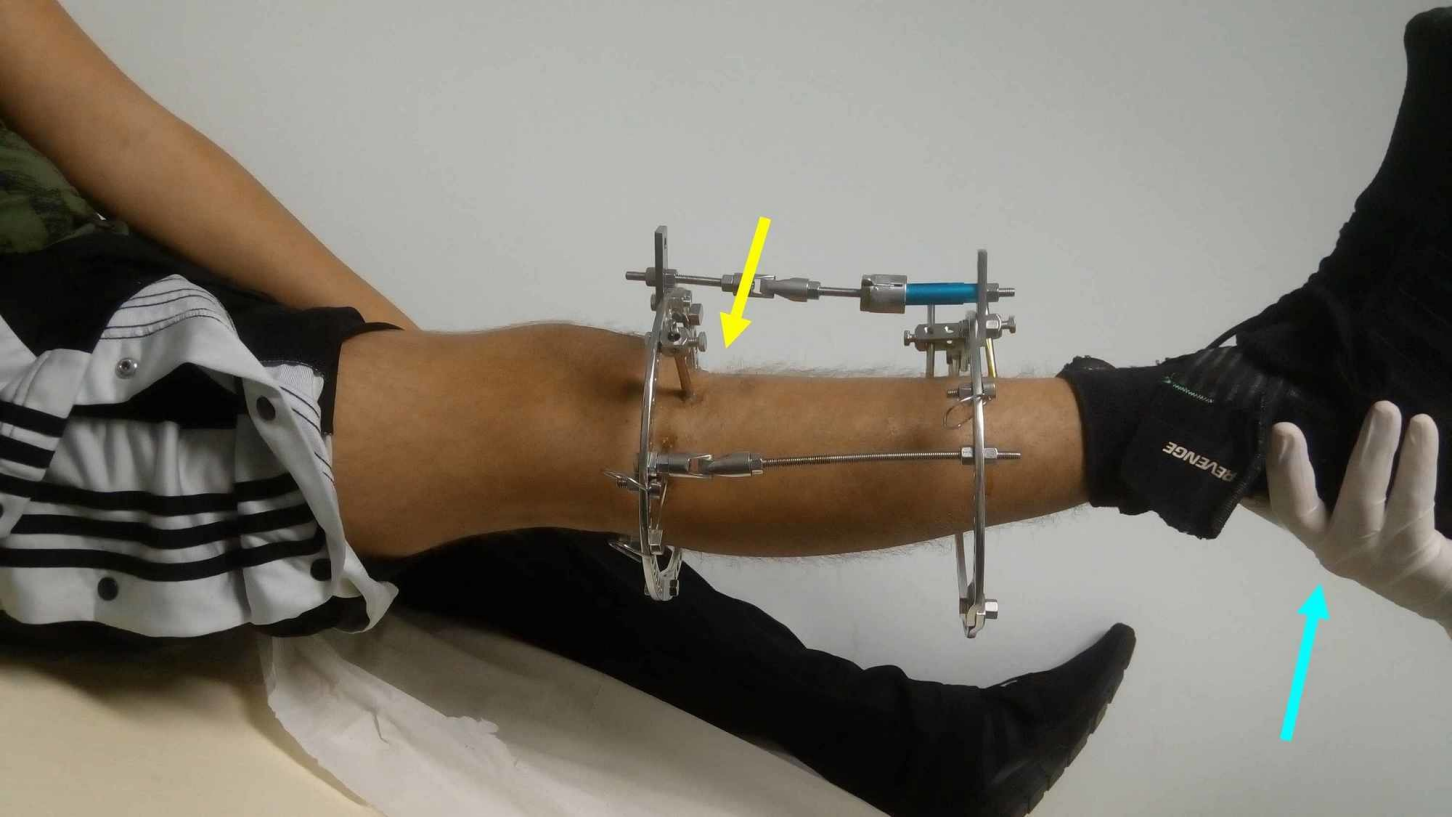
The patient at the 45th postoperative day Complete clinical correction of the deformity (yellow arrow) is observed after heel elevation (blue arrow) of the right leg.

After an initial delay of one week, gradual opening of the osteotomy at a rate of 1 mm per day, in four equal increments, started. Around the 45th postoperative day the desired correction had been reached and the frame was fixed. The patient was observed on a monthly basis and was gradually allowed to bear weight (Figure 5).

**Figure 6:**
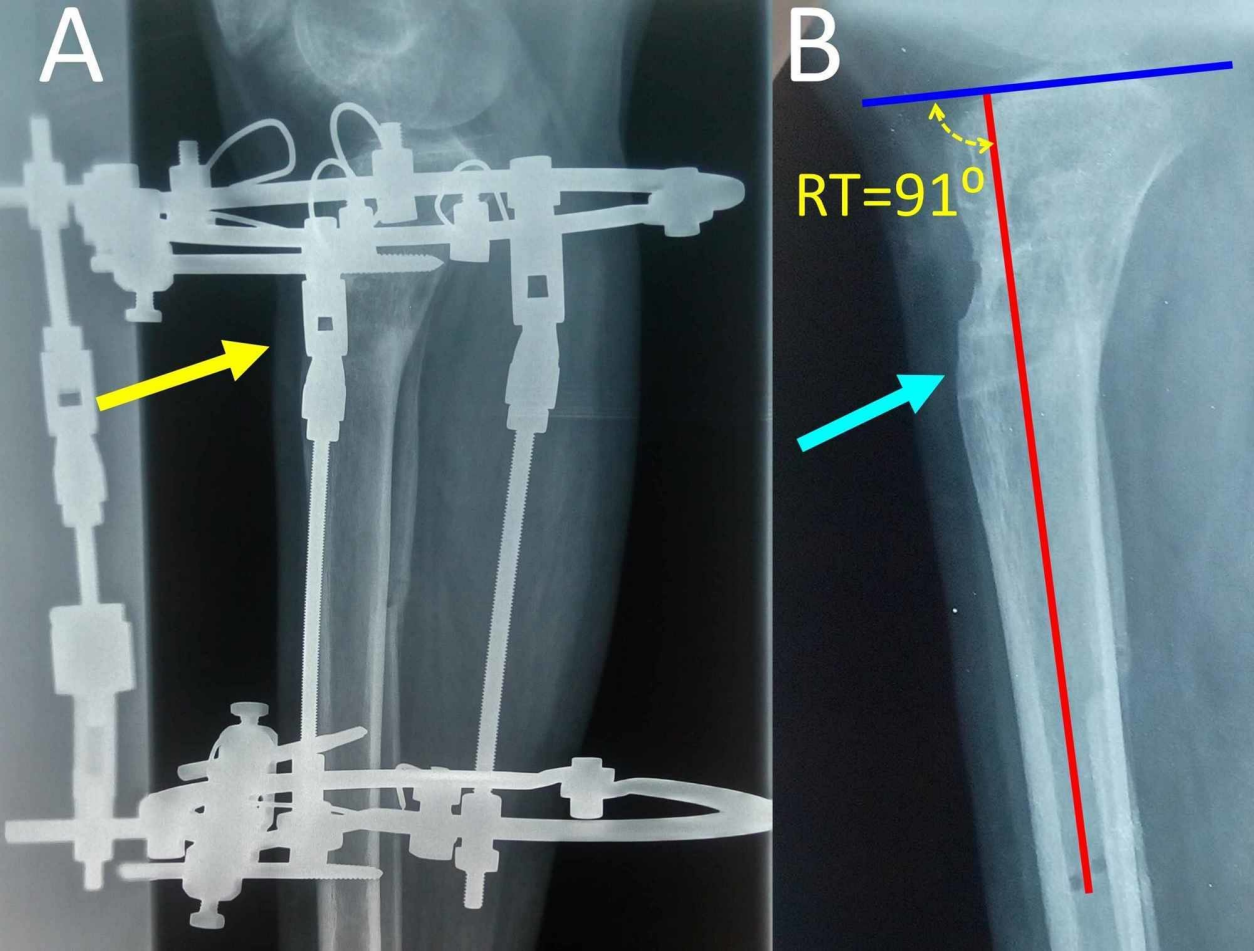
Callus maturation and frame removal at the 120th postoperative day A. Lateral x-ray view of the right tibia: complete ossification of the opening-wedge osteotomy (yellow arrow). B. Lateral x-ray view of the right tibia after frame removal: complete ossification of the osteotomy (blue arrow). The RT-angle is 91⁰, indicating a 30⁰ correction of the osseous deformity. Red line: the distal tibial axis, blue line: the plane of the tibial plateau.

**Figure 7:**
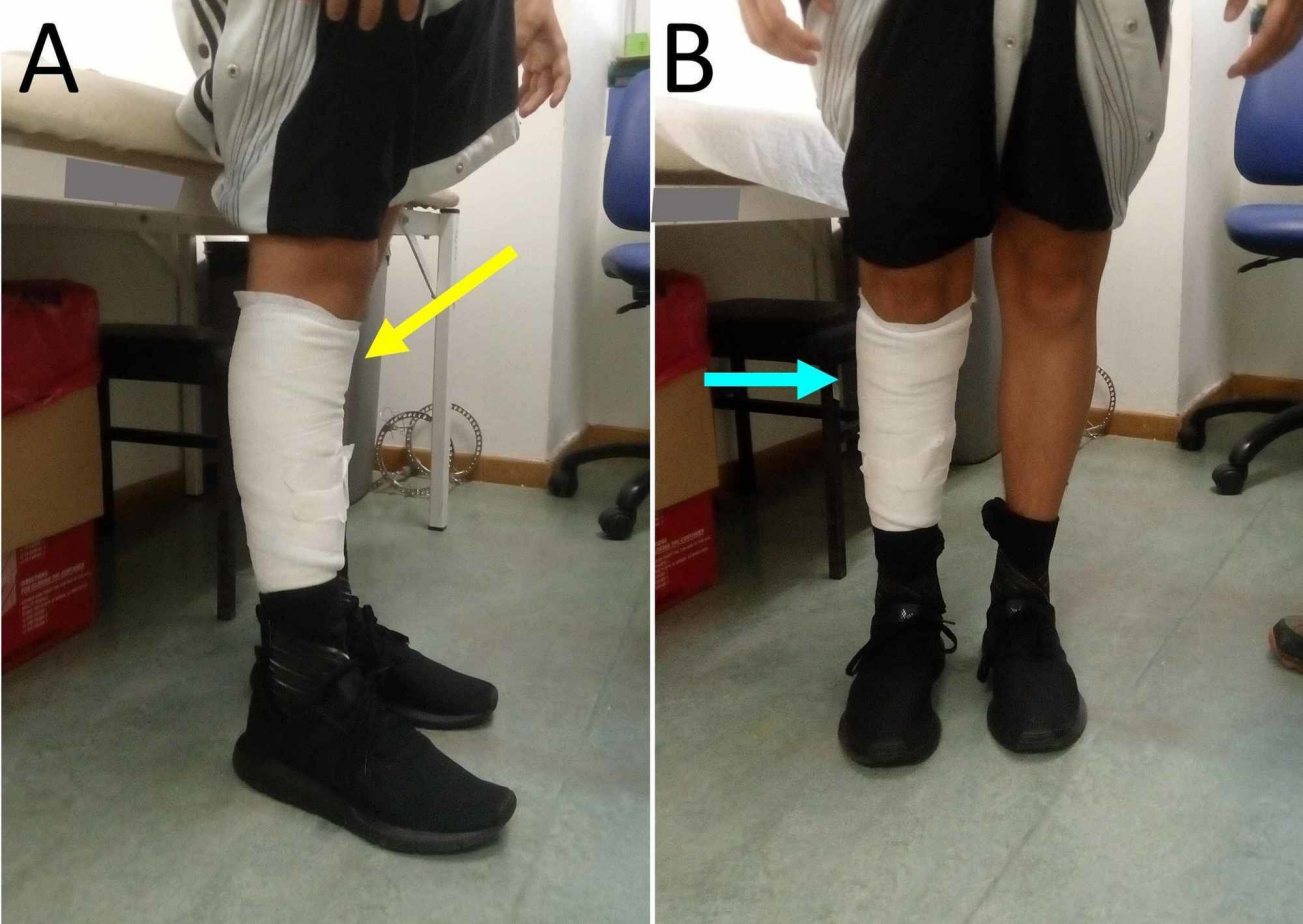
The patient after frame removal A. Complete clinical correction of the recurvatum deformity of the right knee (yellow arrow), B. No obvious valgus deformity of the right knee after frame removal (blue arrow).

Callus maturation completed uneventfully after approximately 120 days postoperatively and the Ilizarof frame was removed (Figures 6, 7). On radiologic examination, the RT-angle was 91⁰. Limb length was equal on clinical examination. The patient’s gait improved markedly. Personal communication with the patient one year postoperatively confirmed an excellent clinical result.

## Discussion

A case of unilateral acquired GR in a 15-year-old boy with the history of GBS a few years before is presented. He had bilateral drop-foot due to permanent paralysis of the muscles of the anterior compartment of the tibia, for which he was wearing below-knee braces for numerous months after recovery from GBS. The braces were fixed with straps on the tibia.

Several cases of GR have a neurologic background. GR has been described in children with poliomyelitis, or in adults after cerebrovascular stroke [17,18]. Weakness of the quadriceps, hamstrings and muscles of the hip, spasticity of the quadriceps, proprioceptive disorders, equinus or drop-foot are among the mechanisms, which favor this deformity in an attempt of the patients to stand and walk [18,19]. Appropriate orthotic devices are of great importance to help compensate motor loss [19].

On the other hand, prolonged immobilization of the lower limb in patients with open growth plates has been accused for early arrest of the proximal tibial growth plate of the tibia [3]. Other authors support, that partial growth arrest of the proximal tibial metaphysis may result from direct pressure on the proximal tibia either from a long-limb cast or from a patellar tendon bearing brace [1,6-9,18-20].

Both factors (neurologic, brace) may have predisposed to the development of GR in the presented case. Repetitive asymmetric loading of the knees, due to his paralytic drop-foot, may have harmed the proximal growth plate of his right tibia, however, the left tibia was spared [20]. On the other hand, the straps used to stabilize the orthosis on the right tibia may have pressed the anterior part of the growth plate and contributed to the osseous GR deformity of the right tibia.

The presented case was successfully treated by means of an anterior opening-wedge proximal tibial osteotomy with gradual distraction using the Ilizarof technique. Distraction stopped when clinical correction was achieved. Further attempt to correct the RT-angle might lead to the opposite result, namely, a flexion deformity of the knee. Furthermore, after frame removal, the straight right lower limb with heel elevation confirmed the pure osseous nature of the GR deformity.

The osteotomy-CORA in this patient was placed distally and dorsally compared to the true-CORA. This allowed simultaneous correction of the angular deformity and gradual lengthening of the anterior cortex, in order to restore lower limb equality. 

Kim et al. described five cases of GR, in which the osteotomy-CORA was placed at the intersection of the bisector of the proximal tibial axis (PTA) and the distal tibial axis (DTA) with the posterior tibial cortex [13]. A quite oblique osteotomy started from below the tibial tubercle and exited at this point. Distraction of the osteotomy to the desired level and filling the gap with autologous iliac bone graft were performed in one stage. The osteotomy was protected with an anatomic locking anteromedial plate. During the procedure the authors report that special care was taken to protect the superficial part of the medial collateral ligament and the posteromedial and posterolateral cortex. The authors report more or less excellent long term results [13]. However, compared with the Ilizarof technique, acute correction of the deformity implies at least two additional surgical procedures: iliac graft harvesting and plate removal. Furthermore, acute stretching of the medial collateral ligament might affect the stability of the knee joint.

In the presented case, selecting distal placement of the osteotomy-CORA relative to the true-CORA had certain benefits. Knee anatomy (tibiofemoral joint, patellofemoral joint) was not affected from the process of correction of the deformity. Intraarticular passage of the tension wires of the proximal Ilizarof ring was avoided, protecting the patient from potentially serious complications, such as inflammation and septic arthritis [16]. The stability of the Ilizarof frame allowed early active motion of the knee. No further surgery to remove the hardware was needed, because the Ilizarof frame was removed on an outpatient basis.

Controversy exists, whether the recurvatum deformity should be measured with the patient standing (weight bearing), or in the supine position [1,2,6,13]. The authors believe that the deformity should be assessed, both clinically and radiologically, with the patient standing. It is thought that weight bearing will reveal the true ligamentous component of the deformity.

## Conclusions

Unilateral acquired GR in a patient with a previous history of Guillain-Barre syndrome and bilateral drop-foot may be the result of the paralytic sequels of this syndrome on the lower limbs. However, GR may also be the result of prolonged bracing of the lower limbs of the patient, in order to deal with drop-foot. Constant pressure by the tibial part of the orthosis at the level of the tibial tuberosity may have caused irreversible damage to the proximal growth plate of the patient's right tibia, leading to early partial growth arrest. A below the tibial tuberosity anterior opening-wedge oblique proximal tibial osteotomy using the Ilizarof technique is a safe treatment option to restore lower limb biomechanics. Proper selection of the center of rotation and angulation of the osteotomy, allows simultaneous correction of the angular deformity and the length deficit of the lower limb, without interfering with knee joint anatomy.
